# Ultra-overt therapy: a novel medical approach centered on patient consciousness

**DOI:** 10.3389/fnint.2024.1457936

**Published:** 2024-08-16

**Authors:** Kamran Shirbache, Amirreza Liaghat, Sanam Saeifar, Ahmadreza Nezameslami, Ali Shirbacheh, Hamid Nasri, Hamidreza Namazi

**Affiliations:** ^1^Department of Orthopedics, Hôpital Robert-Debré, Paris, France; ^2^Immunology from Concepts and Experiments to Translation, CNRS UMR 5164, Université Bordeaux Montaigne, Bordeaux, France; ^3^Buchmann Institute for Molecular Life Sciences (BMLS), Cluster of Excellence Frankfurt Macromolecular Complexes (CEF-MC), Goethe University Frankfurt am Main, Frankfurt am Main, Germany; ^4^Department of Orthopedics, Mayo Clinic, Rochester, MN, United States; ^5^Centre Hospitalier de l’agglomération de Nevers, Nevers, France; ^6^Nickan Research Institute, Isfahan, Iran; ^7^Department of Medical Ethics, School of Medicine, Medical Ethics and History of Medicine Research Center, Tehran University of Medical Science, Tehran, Iran

**Keywords:** overt treatment, mind–body medicine, interoceptive awareness, placebo response, personalized medicine, intersubjective therapy, meaning system, neuroscience

## Abstract

Within the realms of human and artificial intelligence, the concepts of consciousness and comprehension are fundamental distinctions. In the clinical sphere, patient awareness regarding medication and its physiological processes plays a crucial role in determining drug efficacy and outcomes. This article introduces a novel perspective on prescription practices termed “Ultra-Overt Therapy” (UOT). A review of current supporting evidence was conducted through a non-systematic search in PubMed and Google Scholar, focusing on concepts such as the “mind–body relationship,” “placebo response,” “neuroscience,” and “complementary medicine.” Our findings, rooted in the mechanisms of the “placebo effect,” the intricacies of “intersubjective therapy,” the potency of “interoceptive awareness,” and other domains of medical science, suggest that UOT holds theoretical promise. Future research endeavors focusing on these areas may elucidate the global impact of this method on medical treatment and patient care.

## Introduction

The topic of “patient awareness” has an impressive impact on how treatments work and how they are given (Tresker, 2022). Research divides treatments into covert (hidden) or overt (open) based on whether patients know about the medicine they are getting. The overt group, who are told about the drug and how it is given, often feel better because of the placebo effect, unlike the covert group who do not know what they are taking (Colloca et al., 2004). Based on this idea, we introduce the concept of “Ultra-overt therapy” (UOT), suggesting that knowing more about the drug and thinking about how it works can make it works better. This idea leads to examining evidence-based methods and practical research studies to determine whether a third category, termed “ultra-overt,” can be added. It is hypothesized that this approach can impact patient treatments. To explore this, we evaluate the role of consciousness and emotion in physiological events within the body and the subsequent placebo response it elicits. The impact of meaning systems on various treatment methods is then discussed, with an overview of relevant neuroscience findings. As practical evidence, several complementary medicine methods are introduced, employing a systematic approach to the body. Finally, three research studies are proposed to assess the hypothesis from different perspectives.

## Importance of consciousness and emotions

The idea of “consciousness” involves being aware of both internal and external experiences, including thoughts, feelings, and sensory perceptions (internal), as well as environmental inputs (external). Consciousness also involves wakefulness (level of alertness contrasting with sleep or unconscious states), attention (focusing on specific stimuli or thoughts), perception (interprets sensory information to form an understanding) and self-reflection (think about oneself and one’s place in the world) (Tresker, 2022). All these phenomena together integrate information across different brain areas, forming a global workspace where information is broadcasted to other cognitive processes, known as the Global Workspace Theory (Baars et al., 2021). This enhanced cognition leads to “insight,” which involves developing a new understanding that enhances self-awareness (Sattin et al., 2021).

In UOT, we aim to promote patient insight into their medication by encouraging them to think deeply about how the drug works in their body and which organs it affects. This process inevitably impacts both the consciousness and emotions of patients in the short and long term and promotes the global workspace of the patient. As these phenomena impose various effects on the body’s physiology at both macroscopic and microscopic levels (Dembski et al., 2021; Candia-Rivera, 2022; Friedman et al., 2023), it is valuable to consider the different representations of these impacts in relation to patient health and drug efficacy. By fostering a deeper understanding of the medication’s mechanism, patients can experience enhanced therapeutic outcomes and improved overall well-being.

Another critical element in this domain is “emotions.” Emotions are complex psychological and physiological states that involve a range of subjective experiences, cognitive processes, and behavioral responses (Lin and Li, 2023). These are closely connected to the body’s physical and temporal surroundings, including visual stimuli, social situations, and factors like body position, muscle tension, touch, and pain (Savard et al., 2024). All these signals rapidly disseminate throughout the body to elicit responses via the autonomic nervous system, impacting organs such as the heart, lungs, stomach, facial muscles, and limbs (Jerath and Beveridge, 2020).

Real-life observations and research indicate that distinct emotions often manifest in specific body parts, such as anxiety in the stomach area and love or sadness in the heart area. Interoceptive pathways, along with neural pathways, help send signals from your organs and body to your brain, with the insular cortex playing a key role (Nummenmaa et al., 2014). For instance, the close relationship between the brain and digestive system, facilitated by nerve pathways, underscores the significant influence of emotions on digestion, with emotions like fear, worry, and disgust slowing down digestion in response to perceived threats. The gut, often referred to as the “second brain” due to its numerous neurons, plays a pivotal role in gut feelings and intuition, with the microbiome contributing to the transmission of emotional signals throughout the body. By attending to these cues, individuals can regulate their emotions and navigate various situations, particularly when confronted with illness (Lin and Li, 2023; Savard et al., 2024).

These pathways let you pay attention to your body’s signals and focus on what’s happening inside you. Recognizing your medication can activate these pathways to bring about a “somatic reappraisal” (Nummenmaa et al., 2014). “Somatic reappraisal” describes processes that arise from focusing on your internal experiences through interoceptive attention. By using cognitive frameworks to understand insights or connections, you can achieve better responses within yourself and with others (Price and Weng, 2021).

Internally, Stimulus-Independent Thoughts (SITs) are mental processes that occur without any external influences. Studies have examined how SITs impact the human body, showing that they take place both when we are at rest and when we are engaged in tasks. Concentrating on the mechanisms of medication within the body is part of UOT, which induces SITs and provides objective effects on the body (Smallwood et al., 2021).

In addition to the somatic reappraisal effect, “embodiment” is another phenomenon resulting from the significance of awareness and emotions in the mind–body relationship. The connection between bodily sensations and emotional responses, often referred to as “embodiment,” serves as a potential avenue to strengthen the efficacy of UOT (Price and Weng, 2021). According to Heidegger’s perspective, the interrelationship between the mind and body involves a dynamic interplay characterized by reciprocal translation and interaction. Sensations experienced within the body and their subsequent impact on emotions elucidate how physical sensations influence emotional states and vice versa (Fugate et al., 2024).

To explore “embodiment” from another perspective, it is essential to consider therapies that involve sustained focus on the body, guiding individuals to pay attention to specific body parts where emotions are felt, and maintaining that focus with mindfulness. This focused attention is known as an “internal experiential space.” The internal experiential space interprets sensory data based on bodily interactions with the environment, leading to the “cognition process” (Price and Weng, 2021). As the extensive impact of the Global Workspace Theory on the body is mentioned before, here we introduce “interoceptive awareness” as a significant phenomenon arising from the interaction between external stimuli and internal cognition (Baars et al., 2021).

“Interoceptive awareness” is about being able to feel and understand what’s happening inside your body, such as your heartbeat, breathing, and digestion. This awareness can significantly impact your body’s functions by helping you manage stress, control your emotions, and improve your overall well-being. By paying attention to your body’s signals, you can better respond to physical and emotional challenges, leading to better health and functioning (Weng et al., 2021). Recent studies show a strong link between interoception and allostasis, which is the process of efficiently managing and regulating emotions (Jerath and Beveridge, 2020; Price and Weng, 2021; Savard et al., 2024). Many complementary medicines are based on this factor, such as meditation, yoga, and breathwork, which are discussed in the related section. However, one worth explaining here in detail is “body scanning exercises” (Simkin and Black, 2014).

One application of “interoceptive awareness” is in body scanning exercises, a type of mindfulness meditation practice. These exercises involve systematically focusing attention on different parts of the body to develop greater awareness of physical sensations. During a body scan, a person mentally scans their body from head to toe, paying close attention to the sensations in each specific part. This technique can lead to various benefits, including stress reduction, emotional regulation, improved sleep, and pain management. By concentrating on specific body parts and altering their relationship with pain, individuals can more effectively manage chronic pain in those areas (You and Ogawa, 2020). Although the scientific basis of this exercise is not yet fully verified (Gan et al., 2022), it offers notable benefits for patients and aligns with the principles of UOT, which aims to cultivate interoceptive awareness in the therapeutic process for targeted organs.

All the aforementioned evidence can be seen as subjective elements, but to consider something more measurable, the placebo effect can be assessed in different domains of medicine.

## From placebo effect to placebo response

Delving into the enigmatic realm of the UOT, clinicians’ experiences shed light on the placebo’s impact on the patient’s physiology. The placebo effect is intricately linked to the brain’s processes of expectation, anticipation, reward, and learning. However, the mechanisms underlying the brain’s generation of the placebo effect largely elude our understanding. Our current understanding is primarily derived from research on pain relief, Parkinson’s disease, oxygen deprivation, and immune and hormonal reactions. Unfortunately, there is not much information available about other conditions like mental health disorders and physical illnesses, which are the main focus of this hypothesis (Benedetti, 2008; Kradin, 2011).

During the examination of the ways in which placebos reduce discomfort, we see that they work by activating a brain circuit that controls pain using natural painkillers called endorphins. Advanced brain imaging techniques such as functional magnetic resonance imaging (fMRI), positron emission tomography (PET), and electroencephalography (EEG), show that placebos affect key brain areas just like real painkillers do. Placebo also activates certain brain receptors that respond to opioids (Benedetti, 2008; Rossettini et al., 2020). This raises the question of whether thinking about the drug could release the same natural painkillers. If we can boost these receptors, it might enhance the effectiveness of the medication when taken.

On a molecular level, the natural painkillers triggered by placebos interact with other substances involved in pain signaling, such as cholecystokinin (CCK), which can inhibit the pain relief from these natural painkillers. Blocking CCK could potentially strengthen placebo-induced pain relief. The balance between CCK and natural painkillers determines the efficacy of placebos for pain relief. Some types of placebo pain relief do not respond to naloxone, a drug that blocks natural painkillers, showing that other substances like endocannabinoids are involved. Dopamine also plays a big role in how placebos reduce pain, with increased dopamine and natural painkiller activity in certain brain areas. These changes caused by the placebo effect are driven by positive expectations and emotions (Benedetti et al., 2005; Crawford et al., 2021; Schatzberg, 2022; Talib Hassan et al., 2023). The same effects could happen during UOT.

In Parkinson’s disease, placebos can trigger responses that help with symptoms. Placebos cause the brain to release dopamine in certain areas, leading to better symptom control. The amount of dopamine released depends on how much improvement the patient expects. Furthermore, learning also plays a big role in how well placebos work for Parkinson’s disease. Placebos do not help new patients with Parkinson’s disease, but they do help after repeated exposure to a drug that treats the condition (Benedetti, 2008). This shows that consciousness and repeated thinking about the treatment can lead to better results with UOT.

The placebo effect in the immune and hormone systems is linked to conditioning. Giving a placebo after a standard treatment can cause immune or hormone responses like that established medication. For example, repeated administration of ciclosporin, with a flavored drink as a conditioned stimulus, can lead to placebo-induced immunosuppressive responses. This is demonstrated by alterations in specific immune markers and cell activity. Likewise, administering a placebo following sumatriptan, a serotonin agonist, raises growth hormone levels and reduces cortisol (glucocorticoids) secretion, replicating the effects of the drug (Sonawalla and Rosenbaum, 2002; Benedetti, 2008; Frisaldi et al., 2020; Musavi et al., 2023).

Some scientists have broadened the scope of the placebo effect, conceptualizing it as a placebo response that is closely intertwined with unconscious processes. The placebo response manifests involuntarily and subconsciously, operating independently of conscious intention. It involves intricate interactions involving the inherent reward mechanisms of the nervous system, as well as deeply ingrained procedural memories and imaginative constructs. Clinical observations suggest that placebo impacts are not influenced by consciousness, cognitive effort, or deliberate intent, highlighting their ‘automatic’ nature. This is associated with the aggregation of imaginal recollections and fantasies, which exert an inherent influence on nervous system functions, including heart rate, respiration, and electrodermal reactions, often occurring spontaneously and without conscious awareness (Stewart-Williams and Podd, 2004; Benedetti et al., 2005; Benedetti, 2008). Imagination plays a key role in UOT, where patients are encouraged to visualize the drug process and its effects within their bodies in a focused manner.

Considering Descartes’ dualism, it is proposed that the placebo response functions as a fundamental mind–body pathway that promotes “salutogenesis.” This concept emphasizes understanding health through the development of internal resources and strengths, rather than focusing solely on risk factors and vulnerabilities (Brown, 2018). Evidence suggests that the placebo response correlates with central nervous system (CNS) functions, which facilitate the simultaneous emergence of positive emotions and physical sensations that govern mental and physical well-being, especially evident during maternal–infant bonding (Kradin, 2004).

“Salutogenesis” highlights the importance of these internal resources in contributing to health and resilience (Mittelmark et al., 2022). By examining the placebo response through this lens, UOT can offer a unique perspective on how the mind–body connection influences health outcomes (Sattin et al., 2021). Given this intrinsic link between CNS development and the placebo response, it is valuable to explore the neuroscience domain in relation to UOT to deepen our understanding of these interactions.

## Neuroscience

Neuroscience integrates knowledge from diverse fields such as psychology, biology, chemistry, and medicine, encompassing various aspects of the nervous system, including neuroanatomy, neurophysiology, neurobiology, and cognitive neuroscience. This integration provides a comprehensive understanding of behavior, cognition, emotions, and sensory experiences (Nebe et al., 2023).

An important aspect of UOT is the impact of the “meaning system” on the body. In human psychology, the “meaning system” refers to the complex network of beliefs, values, expectations, and interpretations that individuals use to make sense of their experiences and the world around them. It encompasses an individual’s understanding of themselves, others, and the broader social and cultural context in which they live. The meaning system significantly influences how people perceive events, attribute meaning to them, and respond emotionally, cognitively, and behaviorally (Schore, 2021; Signorelli and Meling, 2021).

Current studies in neuroscience using brain imaging techniques to explore how the brain functions during meaning system process. Researchers are applying machine learning to brain scan data to understand how different cognitive states, such as focused breathing or daydreaming, affect brain activity. This research helps scientists observe how attention shifts during meditation, particularly involving the Prefrontal Cortex, which is active during meditation, focused breathing, and daydreaming (Wang et al., 2023). Imagining how a drug works in the body through mental visualization—an approach used in UOT—can be seen as a form of meditation or daydreaming, potentially sharing similar mechanisms (Bechtel and Huang, 2022). Interestingly, the Prefrontal Cortex is also implicated in Global Workspace Theory (Baars et al., 2021).

Our brain’s Default Mode Network (DMN) becomes active when we engage in self-referential thinking or reflect on our thoughts. The anterior insula, part of the Salience Network, plays a crucial role in maintaining awareness of bodily states and facilitating communication between different brain networks. It aids in processing significant events and regulating emotions, often without our conscious awareness, such as when we recall past stressful experiences (Buckner et al., 2008). The DMN includes several key regions: the Posterior Cingulate Cortex (PCC), Precuneus, Angular Gyrus, and Medial Prefrontal Cortex (mPFC). Notably, the mPFC, which is involved in the DMN, also activates during meditation (Signorelli and Meling, 2021; Wang et al., 2023).

On the other hand, some scientists propose that the brain evolves from a foundational neural network established during gestation. This initial neural framework undergoes further development through repetitive stimulation, leading to the formation of additional, interconnected neural networks. These networks engage in temporally linked re-entrant communication, facilitated by components of the secondary neural repertoire, which generates complex brain functions such as perception and consciousness. A discernible psychophysical “neurosignature” emerges from the organized activities of these secondary neural networks. Within this framework, the sense of self develops, encompassing both unique psychological traits and automatic, implicit procedural actions that regulate bodily functions such as metabolism, autonomic balance, and musculoskeletal alignment (Kradin, 2004; Bechtel and Huang, 2022). These robust networks underscore the potential effects of UOT on health by integrating various cognitive and emotional processes.

To explore neuroanatomy further, the amygdala plays a crucial role in our emotional responses by processing sensory information and triggering emotions. It helps us recognize threats and regulate our reactions (Nummenmaa et al., 2014). The amygdala communicates with other brain regions, such as the prefrontal cortex and hippocampus, to coordinate our emotions and behaviors with hormonal signals. Additionally, it releases stress hormones, such as cortisol, in response to perceived threats. Our level of consciousness can influence how hormones and receptors interact, as well as the feedback loop between them (Davis and Whalen, 2001; Bechtel and Huang, 2022). Similarly, UOT can facilitate the molecular and cellular interactions of drugs within targeted organs through analogous neural mechanisms.

## Complementary medicine and other approaches

While Western medicine primarily examines how the body’s physical pathways influence consciousness, emotions, symptoms, and responses to placebos, the enigmatic mechanisms of complementary methods may also contribute to defining UOT (Orkin, 2021). Although there is no definitive scientific evidence supporting the efficacy of alternative therapies such as energy healing, acupuncture, yoga, and homeopathy, these approaches emphasize the constructive impact of awareness on the mind–body relationship and its potential to aid the healing process (Tangkiatkumjai et al., 2020; Urits et al., 2021).

For instance, many of these therapies focus on specific energy points in the body, such as chakras and Qi, to promote well-being (Khalsa, 2007; Simkin and Black, 2014; Venkatesh et al., 2020; Rogers et al., 2021). Homeopathy, in particular, operates on the belief in a vital force that connects all parts of the body and seeks to enhance this force to restore health. According to this theory, thoughts and emotions can influence this vital force, with positive thoughts strengthening it and negative thoughts weakening it (Mathie, 2015; Aversa et al., 2016). Although these methods have not been fully validated as scientific practices, their historical use and some theoretical mechanisms discussed in literature suggest potential benefits (Adhikari et al., 2018; Deuel and Seeberger, 2020; Urits et al., 2021; Gan et al., 2022).

Turning back to conventional medicine, several traditional approaches can shed light on UOT. For example, “Narrative Medicine” highlights the importance of effective communication with patients. This approach uses storytelling and narrative techniques in clinical practice, medical education, and healthcare research to improve healthcare delivery by incorporating the narratives of both patients and healthcare providers (Remein et al., 2020).

By prioritizing the patient’s story, Narrative Medicine acknowledges that illness experiences are deeply personal, shaped by individual life stories, values, beliefs, and cultural backgrounds. Integrating literature, poetry, film, art, and reflective writing into medical education helps healthcare providers develop empathy, creativity, and moral imagination (Loy and Kowalsky, 2024). Given that imagination plays a crucial role in UOT, the principles of Narrative Medicine can also help explain and support the UOT framework.

Another example of the significance of communication in medical practice is the concept of “phenomenology” of disease. This approach allows scholars to delve into patients’ lived experiences, offering fresh insights into health, illness, and healthcare delivery. “Medical Phenomenology” encourages both doctors and patients to reflect on their interactions, exploring moments of connection, misunderstanding, resistance, and growth (Fernandez, 2020). This reflection can lead to a form of “inter-subjective therapy” a psychotherapeutic approach that emphasizes the relational and interactive aspects of the therapeutic process (Stanier and Miglio, 2021).

Inter-subjective therapy centers on the therapeutic relationship between the therapist and the patient, asserting that our sense of self and reality is shaped by our interactions with others. By focusing on the process rather than solely on outcomes, inter-subjective therapy prioritizes understanding and exploring the therapeutic journey within the individual’s body (Stanier and Miglio, 2021; Weng et al., 2021).

Delving deeper, the philosophical foundation of “Hermeneutic Medicine” emphasizes the importance of interpretation, dialogue, and contextual understanding in clinical practice, particularly focusing on “meaning-making in diagnosis and treatment.” This approach encourages healthcare providers to interpret patients’ symptoms and diagnostic results within the context of their unique personal narratives and circumstances, guiding more personalized treatment (Svenaeus, 2022).

Inter-subjective therapy aligns with this perspective by highlighting the relational and interactive aspects of the therapeutic process. Although primarily used for psychological conditions, it can also enhance self-awareness and insight into the healing process when integrated with UOT. By fostering a deeper understanding of the patient’s experiences and the therapeutic process, this combined approach can create meaningful engagement, promote transformative change, and ultimately support the healing journey (Stanier and Miglio, 2021; Trevarthen et al., 2023).

If we define UOT as a ritual in which the patient watches or reads about the treatment carefully and then each time thinks about the process of treatment inside his/her body with concentrated focus, it is valuable to consider “Ritual Medicine” and the related literature as a similar approach in medicine.

“Ritual medicine” represents a realm yet to be fully explored, delineating two fundamental roles of rituals in the therapeutic process: an expressive function and a creative function. In its expressive dimension, a ritual communicates essential values and cultural paradigms through symbolic representation, theatrically articulating these foundational principles and imparting them to both active participants and observers (Dallas et al., 2020). Conversely, in its creative capacity, rituals actively shape or reestablish frameworks through which individuals perceive reality, encompassing societal structures, and ethical principles. Rituals, characterized by their performative, standardized, and metaphorical nature, endeavor to translate thoughts into actions, bridge the gap between self and others, and reconcile historical narratives with present experiences (Goli and Farzanegan, 2016).

It is evident that rituals, serving as therapeutic symbolic expressions across verbal, physical, and spatial dimensions, possess the potential to alter focus, memory, and expectations of recovery, thereby instilling a profound sense of potential well-being. Experimental investigations into the configurations and significance of ritual symbols, particularly those intertwined with healing ceremonies, have confirmed that each symbol carries a multitude of connotations for participants, illuminating societal values, organizational structures, and perspectives on natural and supernatural domains. The interpretative framework, preparatory stage, and enactment of a ritual can significantly modulate health perceptions, behaviors, and psychoneuroimmunological reactions through both direct and indirect implications. A significant aspect of this effect is attributed to the emotions elicited by rituals, which harmonize internal, interpersonal, and transpersonal spheres (Goli and Farzanegan, 2016; Apud and Romaní, 2020).

In addition to the guidance provided in the UOT, which instills a sense of ritualistic efficacy in patients, it fosters confidence during treatment, cultivating belief in its effectiveness and ultimately yielding more favorable outcomes imbued with positive emotions (Goli and Farzanegan, 2016; Apud and Romaní, 2020).

Now that we have discussed every element that could be important in the interaction of medicine on a molecular-cellular level in our body relating to the mechanism of UOT, it is time to consider the systemic factors that help achieve better results in understanding UOT.

## Systemic factors

To consider the systemic factors in UOT, it is important to think about how drugs distribute in the body and how they are delivered to certain areas more than others at different times. This is influenced by the activation of the sympathetic nervous system (SNS) and the inactivation of the parasympathetic nervous system (PNS), along with the release of certain chemicals like nitric oxide, prostaglandins, and adenosine, which lead to vasodilation (Gibbons, 2019). While the SNS and PNS regulate the involuntary distribution of blood to various parts of the body, a person can influence this distribution intentionally through mental focus, meditation, and biofeedback, as explained above. It is hypothesized that this intentional increase in blood delivery to a specific organ can be achieved consciously or unconsciously through UOT (Kreibig, 2010).

If we consider humans as a combination of genotype and phenotype, “personalized medicine” can also play an additive role in UOT. Personalized medicine customizes medical treatments and interventions according to individual characteristics, such as genetic makeup, lifestyle factors, and environmental influences, to optimize patient outcomes. It selects the most effective and suitable treatments based on each individual’s unique traits (Whirl-Carrillo et al., 2021).

The UOT can serve as another manifestation of this scientific approach. Genetic testing, biomarker testing, pharmacogenomics, and patient engagement, which form the foundation of personalized medicine, are all encompassed within the “ultra-overt” method (Hassan et al., 2022). This is because eliciting each patient’s consciousness is intertwined with all these parameters individually.

All the ways mentioned above show the immediate effects of consciousness on medication. The long-term effects of the placebo response can vary depending on the person and the condition being treated. Research on how genetics play a role in the placebo effect is still new, and unknown factors may also correlate with UOT (Hassan et al., 2022) (Table 1).

**Table 1:** Supporting evidence for the effectiveness of ultra-overt therapy.

Consciousness	Interoceptive awareness (You and Ogawa, 2020) Embodiment (Fugate et al., 2024) Somatic reappraisal (Price and Weng, 2021) Stimulus-independent thoughts (Smallwood et al., 2021)
Placebo effect	Activating brain areas and related receptors (Benedetti et al., 2005; Rossettini et al., 2020) Secretion of agonists and antagonists (Benedetti, 2008) More efficacy in repeated exposure (Frisaldi et al., 2020) Link to conditioning (Sonawalla and Rosenbaum, 2002)
Neuroscience	Commonalities in neural pathways and neurotransmitters (Signorelli and Meling, 2021) Activity of the prefrontal cortex in both cognition, emotional and physiological processes (Wang et al., 2023) Default mode network (Buckner et al., 2008) Neurosignature (Bechtel and Huang, 2022) Role of amygdala (Davis and Whalen, 2001)
Complementary medicine and other approaches	Energy healing, acupuncture, yoga, meditation and homeopathy (Aversa et al., 2016; Urits et al., 2021) Narrative medicine (Remein et al., 2020) Inter-subjective therapy (Stanier and Miglio, 2021) Hermeneutic medicine (Svenaeus, 2022) Ritual medicine (Goli and Farzanegan, 2016)
Systemic factors	Drug distribution associated with autonomic nervous system (Kreibig, 2010) Personalized medicine (Whirl-Carrillo et al., 2021) Genetics (Hassan et al., 2022)

## Clinical study

To test the “ultra-overt” hypothesis, we suggest three research plans for further investigation in this case.

First, to make the ultra-overt treatment work best for patients, we need to educate them fully about the drug’s target organ and how it works on a molecular level. We can show them an animation of the drug delivery process and its effects on the tissue, receptors, and organ. To measure the impact, we ask them to watch the animation each time they take the medicine.

## First project: amlodipine

One way the ultra-overt method can work is by affecting distribution. To study this, we can use amlodipine to expand its effect on calcium channels in the heart and blood vessels to lower blood pressure, as well as in the bowel muscles which can cause constipation. To see how amlodipine interacts with calcium channels in the heart, blood vessels, and bowel, we can use patch-clamp electrophysiology or radioligand binding assays (Li et al., 2022).

In the first phase, we check the calcium channels in all targeted organs of the patient after amlodipine treatment for a certain time. Then, in the second phase, we inform the patients about amlodipine’s effect on heart muscles and show them an animation focusing on how the drug works in the heart to control blood pressure. We then measure the interactions between amlodipine and calcium channels in all organs of the same patients (including blood vessels and bowel) and compare the results to check if knowing about amlodipine’s impact on the heart causes a significant change in how it interacts with calcium channels compared to the blood vessels and bowel before and after the intervention.

## Second project: metformin

To study the impact on organs, we can use a drug like metformin that targets two organs with different mechanisms. Metformin reduces glucose production in the liver by blocking a pathway called AMP-activated protein kinase (AMPK), and it also makes tissues more sensitive to insulin by improving insulin signaling and glucose uptake in muscles and fat tissue. By looking at these pathways separately, we can see how metformin can affect liver glucose production and insulin sensitivity in muscles by measuring glycogen storage in the liver and muscles using MRI (Shuai et al., 2020; Nourmohammadi et al., 2023).

Like the first project, we check the glycogen storage in the liver and muscles during metformin treatment for a certain time before the ultra-overt education. In the second phase, we tell the patients about metformin’s effect on the liver and show them an animation focusing on how the drug works in the liver to reduce glucose production. We then measure the glycogen storage in both targets (liver and muscles) to determine if the patients’ awareness of the impact of metformin on the liver causes a significant change in glycogen storage in the liver compared to the muscles before and after the intervention.

## Third project: bupropion

This idea can work on a cellular and molecular level by affecting receptors and modulators. Bupropion acts as a norepinephrine-dopamine reuptake inhibitor and a nicotinic acetylcholine receptor blocker, commonly used for depression and quitting smoking. This drug can increase levels of norepinephrine and dopamine in the brain and block nicotinic acetylcholine receptors (Schatzberg, 2022). By studying the patients like the first project and informing them only about bupropion’s role in increasing norepinephrine and dopamine, we can measure the increment in these chemicals and the blockage of nicotinic acetylcholine receptors in the brain using PET imaging or radiolabeled ligands before and after the intervention (Gharibkandi et al., 2020). By comparing the changes in dopamine/norepinephrine levels with the change in nicotinic acetylcholine receptor blockage, we can see how knowing about the drug’s molecular mechanism affects the function of the drug in the body.

It is important to note that we should seek the “correlation” between the effect of the ultra-overt technique intervention and the final outcome in drug efficacy, rather than focusing on “causation.” As mentioned earlier, this technique involves multiple domains and factors within the body, necessitating a comprehensive analysis of the outcomes (Schellenberg, 2020).

## Conclusion

In conclusion, the “Ultra-Overt Therapy” (UOT) endeavors to engender “interoceptive awareness” in patients regarding the medications they are using for their ailments and the subsequent physiological responses within their bodies. It is evident that this technique may yield diverse effects depending on the nature of the illness, whether acute or chronic, somatic or psychiatric, and the specific organs involved. As certain aspects of the underlying mechanisms of the placebo effect remain enigmatic, additional clinical investigations are imperative to ascertain the efficacy of UOT comprehensively.

## Data availability statement

The original contributions presented in the study are included in the article/supplementary material, further inquiries can be directed to the corresponding author.

## Author contributions

KS: Conceptualization, Writing – original draft, Writing – review & editing. AL: Investigation, Writing – original draft. SS: Validation, Writing – review & editing. AN: Investigation, Resources, Writing – review & editing. AS: Validation, Writing – review & editing. HNas: Project administration, Supervision, Writing – review & editing. HNam: Conceptualization, Supervision, Writing – review & editing.
